# Solo emergency care by a physician assistant versus an ambulance nurse: a cross-sectional document study

**DOI:** 10.1186/s13049-016-0279-3

**Published:** 2016-06-29

**Authors:** Anneke Bloemhoff, Lisette Schoonhoven, Arjan J. L. de Kreek, Pierre M. van Grunsven, Miranda G. H. Laurant, Sivera A. A. Berben

**Affiliations:** Eastern Regional Emergency Healthcare Network, Radboud University Medical Centre, PO Box 9101, 6500 HB Nijmegen, Netherlands; Faculty of Health Sciences, University of Southampton, Southampton, UK; Scientific Institute for Quality of Healthcare, Radboud University Medical Centre, Nijmegen, Netherlands; Ambulance Emergency Medical Service Veiligheids- en Gezondheidsregio Gelderland-Midden, Arnhem, Netherlands; Ambulance Emergency Medical Service Veiligheidsregio Gelderland-Zuid, Nijmegen, Netherlands; Department of Organisation of Health and Services, Faculty of Health and Social Studies, HAN University of Applied Sciences, Nijmegen, Netherlands; Department of Emergency and Critical Care, Faculty of Health and Social Studies, HAN University of Applied Sciences, Nijmegen, Netherlands

**Keywords:** Physician assistants, Paramedical personnel, Emergency medical services, Ambulances, Prehospital emergency care, Nursing, Netherlands

## Abstract

**Background:**

This study compares the assessment, treatment, referral, and follow up contact with the dispatch centre of emergency patients treated by two types of solo emergency care providers in ambulance emergency medical services (EMS) in the Netherlands: the physician assistant (PA), educated in the medical domain, and the ambulance registered nurse (RN), educated in the nursing domain. The hypothesis of this study was that there is no difference in outcome of care between the patients of PAs and RNs.

**Methods:**

In a cross-sectional document study in two EMS regions we included 991 patients, treated by two PAs (*n* = 493) and 23 RNs (*n* = 498). The inclusion period was October 2010-December 2012 for region 1 and January 2013-March 2014 for region 2. Emergency care data were drawn from predefined and free text fields in the electronic patient records. Data were analysed using descriptive statistics. We used *χ*^*2*^ and Mann-Whitney U tests to analyse for differences in outcome of care. Statistical significance was assumed at a level of *P* <0.05.

**Results:**

Patients treated by PAs and RNs were similar with respect to patient characteristics. In general, diagnostic measurements according to the national EMS standard were applied by RNs and by PAs. In line with the medical education, PAs used a medical diagnostic approach (16 %, *n* = 77) and a systematic physical exam of organ tract systems (31 %, *n* = 155). PAs and RNs provided similar interventions. Additionally, PAs consulted more often other medical specialists (33 %) than RNs (17 %) (χ^*2*^ = 35.5, *P* <0.0001). PAs referred less patients to the general practitioner or emergency department (50 %) compared to RNs (73 %) (χ^*2*^ = 52.9, *P* <0.0001). Patient follow up contact with the dispatch centre within 72 h after completion of the emergency care on scene showed no variation between PAs (5 %) and RNs (4 %).

**Conclusions:**

In line with their medical education, PAs seemed to operate from a more general medical perspective. They used a medical diagnostic approach, consulted more medical specialists, and referred significantly less patients to other health care professionals compared to RNs. While the patients of the PAs did not contact the dispatch centre more often afterwards.

## Background

Worldwide, there is an increasing demand for ambulance emergency medical services (EMS) in developed countries [1]. The continued rise in utilisation of emergency ambulances leads also to an increasing demand on the wider health care system, e.g. emergency departments (EDs), out-patient clinics, and acute hospitals. Potentially, it compromises access, quality, safety, costs, and outcomes of emergency care. Today, it is unclear what is the optimal care provision in prehospital care, and how prehospital care can be effective; not only in diagnostics and treatment, but also in triaging patients for the right level of care, from a socioeconomically perspective.

In the Netherlands, prehospital emergency care is regularly provided by ambulance registered nurses (RN) [2] as solo emergency care providers, and emergency medical teams, driver and RN, using a full size equipped ambulance. Recently, the position of the physician assistant (PA) has been introduced in ambulance EMS in the Netherlands. The PA works as solo emergency care provider. The aim of introducing a skill mix of PA and RN into ambulance EMS is twofold. First, EMS organisations want to assess and treat patients with emergency care complaints more adequately, as PAs work in the medical domain and RNs in the nursing domain. And second, the regional EMS organisations are exploring opportunities for individual growth and career perspectives of RNs through the PA education.

The education and competences of RNs and PAs differ. The RN has a mandatory 4 year bachelor education to become a registered nurse. Most RNs follow an education in intensive care, emergency care and/or anaesthesiology, before they become an ambulance RN. Each of these supplementary nursing courses are combined with practice learning and take 18 up to 24 months of training, depending on a full or part time employment. Furthermore, the RN follows a prehospital ambulance emergency care education of 7 months, which is mainly focused on stabilisation of vital signs, early interventions, and the prevention of relapse and adverse events. The PA, however, has a 4 year bachelor education in health care, followed by a 30 month medical training programme at a master level. A substantial part of the PA education is focused on medical/diagnostic competences and skills, such as examination of the patients’ organ tract systems [3].

The RN is registered as a nurse according to the Dutch Healthcare Performance Act [4], and has a functional autonomy within the framework of the national EMS standard. This standard covers 113 flowcharts with decision making strategies on diagnosis and treatment of signs and symptoms of 15 diagnosis groups e.g. airway, cardiology, internal medicine and trauma care. The professional autonomy of the PA in the Netherlands is comparable to the autonomy of a doctor of medicine without any medical specialisation [4]. Furthermore, there are no specific national EMS standards for the PA.

The literature describes that the PA is increasingly involved in primary care teams [5, 6] and at the emergency department (ED) [7]. However, we found no information on the role and function of the PA in ambulance EMS. Therefore, the aim of this study was to describe the patient care of the PA and RN as solo emergency care providers, based on differences in education. The hypothesis of this study was that there is no difference in outcome of care between the patients of PAs and RNs.

## Methods

### Study design

We performed a cross-sectional document study to provide insight in the patient care of the PA and RN, working as solo emergency care providers in EMS. On the basis of the study protocol, the Committee on Research Involving Human Subjects region Arnhem/Nijmegen waived the need for ethical approval (registration number 2016–2355).

### Population and setting

Patients with urgency level A1 (arrival <15 min) and A2 (arrival <30 min) were enrolled in the study. We included all patients treated by two PAs in the inclusion period of two EMS organisations in the Netherlands: EMS Veiligheids- en Gezondheidsregio Gelderland-Midden (VGGM, *n* = 1) and EMS Veiligheidsregio Gelderland-Zuid (VRGZ, *n* = 1). For each EMS organisation we randomly selected, by syntax command in SPSS, an equal number of patients who were treated by RNs as solo emergency care providers: VGGM (*n* = 12 RNs) and VRGZ (*n* = 11 RNs). Due to a different employment date of the PAs in VRGZ and VGGM, the inclusion period for VRGZ was October 2010-December 2012 and for VGGM January 2013-March 2014. The inclusion number of PAs, RNs, and their patients was based on feasibility and not on a formal power analysis.

### Data collection

Data were drawn from the EMS Electronic Patient Records (EPR). Unique patient identifiers were excluded or made anonymous. We performed the data extraction according to a standardized protocol developed by PvG, SB, AdK, LS, see Table 1. Predefined data in the EPR were directly extracted. Other data were extracted from free text notes, interpreted, and categorised by two independent researchers (BvdA, PS). The first 100 cases were cross-checked. The latter data extraction was supervised and double-checked by a third researcher (AdK).

**Table 1:** Data collection of key outcome measures for solo emergency physician assistants (PA) and ambulance nurses (RN) or PA specific

Data collection method	Key outcome measure (PA, RN)	Data processing protocol
Data extracted from predefined fields in the EPR^a^	Age categories (PA, RN)	Run date minus birth date
	Gender (PA, RN): male, female	NP^b^
	Level of urgency according to Dutch EMS triage standard (PA, RN): A1: ‘very urgent’ complaints needing arrival on scene within 15 min; A2: ‘urgent’ complaints needing arrival on scene within 30 min	NP^b^
	Monitoring (PA, RN): Respiratory rate, Oxygen saturation, Systolic blood pressure, Diastolic blood pressure, Pulse rate, ECG^c^/heart rhythm, GCS/AVPU^d^, Glucose, Body temperature, Pain intensity score: ‘yes’, ‘no’	When vital sign measurement was registered in EPR^a^: variable was coded ‘yes’
	Interventions described in the national EMS standard (PA, RN): Placement of intravenous drip, Supply of oxygen, Immobilisation: ‘yes’, ‘no’	When intervention was registered in EPR^a^: variable was coded ‘yes’
	Administer medication according to EMS standard (PA, RN): ‘yes’, ‘no’	When medication, included in EMS standard, was registered in EPR^a^: variable was coded ‘yes’
	Administer medication not in national EMS standard (PA): ‘yes’, ‘no’	When medication, not included in EMS standard, was registered in EPR^a^: variable was coded ‘yes’
	Referral after EMS treatment (PA, RN): ‘yes’, ‘no’	NP^b^
	Type of healthcare organisation referred to (PA, RN): General Practitioner, Emergency Department	NP^b^
	Treatment time on scene (minutes) (PA, RN)	Time of departure minus time of arrival
	Follow up contact after prehospital EMS care within 72 h and within 24 h (PA, RN): ‘yes’, ‘no’	Identification of registered additional call to dispatch centre within 72 h and within 24 h after time of departure from scene
Data based on free text notes	Initial complaints or conditions (PA, RN): trauma, non trauma, deceased	Free text notes were identified and categorized according to these conditions
	SCEBS^e^ methodology used (PA): ‘yes’, ‘no’	When free text notes were ordered according to SCEBS^e^ methodology: variable was coded ‘yes’
	Systematic physical exams of organ tract systems (PA): ‘tractus pulmonalis’, ‘circularis’, ‘abdominalis’, ‘neurology’, ‘extremities’, ‘gynaecology’, ‘urogenitalis’, ‘ear-nose-throat’ Systematic physical exams of organ tract systems (PA): ‘yes’, ‘no’	Free text notes were identified and categorized according to these tracti; When one or more tracti were identified: variable was coded ‘yes’
	Interventions not described in the national EMS standard (PA): Suture, Medical advice to patient: ‘yes’, ‘no’	When intervention was identified in the free text notes: variable was coded ‘yes
	Interventions not described in the national EMS standard (PA): Consultation of health care professional regarding referral: ‘consultation of General Practitioner’, ‘consultation of Emergency Department’, ‘consultation of other health care professional’, ‘no consultation’	Free text notes were identified and categorized according to these type of health care professionals

^a^ *EPR* electronic patient records, ^b^ *NP* no additional processing needed, ^c^ *ECG* electrocardiograph, ^d^ *GCS/AVPU* Glasgow Coma Scale/Alert Voice Pain Unresponsive, ^e^ *SCEBS* Somatic complaints, Cognitions, Emotions, Behaviour, and Social functioning of the patient

### Key outcome measures

Key outcome measures are shown in Table 1, together with the type of source, predefined field or free text notes, and the data processing protocol. First, we identified patient characteristics, level of urgency defined according to the Dutch EMS triage criteria [8] and the initial complaints or conditions of the patient (trauma [9] or non-trauma).

Second, we identified diagnostic measurements and interventions provided to the patient by PAs and RNs, according to their education and described in the national EMS standard. In the free text notes of the EPR, we identified the use of a systematic medical diagnostic approach, the SCEBS methodology (focused at Somatic complaints, Cognitions, Emotions, Behaviour, and Social functioning), according to the PA education. Furthermore, we examined whether PAs performed systematic physical examination of the organ tract systems, such as pulmonary tract, circulatory tract. Additionally, we extracted data on interventions provided by PAs and RNs based on their education. We classified these as described or not described in the national EMS standard. Finally, we examined the outcomes of patient care in terms of consultation of other medical specialists, referral pattern, length of treatment time on scene and follow-up contacts with the dispatch centre within 24 and 72 h after completion of treatment on scene.

### Analytical methods

Data were analysed using descriptive statistics. In case of missing data only valid data were used in the descriptive statistics. The Tables show the total and the valid numbers. We used χ^*2*^ and Mann-Whitney U test to analyse for similarities and differences in outcome of patient care between PAs and RNs. Statistical significance was assumed at a level of *P* <0.05. All statistical analyses were carried out using Statistical Package for Social Sciences (SPSS version 22).

## Results

In total 991 EMS runs were included in the study, 493 patients were treated by the PA (*n* = 2) and 498 patients were seen by the RN (*n* = 23). Nineteen runs were excluded from the study because they were not emergency A1 or A2 runs (*n* = 15), or they were registered twice (*n* = 4).

### Patient characteristics, initial conditions and triage

The mean age of the patients was 50 years (PA: 52 (SD 25), *n* = 462; RN: 48 (SD 24), *n* = 496), and half of the patient sample was male (PA: 53 %, *n* = 251; RN: 51 %, *n* = 252). Two out of five initial conditions of the patients were trauma related (PA: 45 %, *n* = 218; RN: 40 %, *n* = 200), e.g. ‘injury due to fall’ (Table 2). Four patients were dead on the time of arrival at the scene (PA: *n* = 3; RN: *n* = 1). Most patients were triaged as ‘very urgent’ (PA: 70 %, *n* = 338; RN: 73 %, *n* = 361) and a smaller proportion as ‘urgent’ (PA: 30 %, *n* = 144; RN: 27 %, *n* = 136).

**Table 2:** Initial complaints and conditions of patients treated by solo emergency physician assistants (PA, *n* = 2) and ambulance nurses (RN, *n* = 23)

Initial complaints/conditions of patients	PA (*n* = 481)^a^	RN (*n* = 495)^b^	Initial complaints/conditions of patients	PA (*n* = 481)^a^	RN (*n* = 495)^b^
*Trauma/Injury* (in alphabetical order, *n* (%))	218 (45 %)	200 (40 %)	*Non trauma* (in alphabetical order, *n* (%))	260 (54 %)	294 (59 %)
Auto mutilation	2		Acute confusion/delirium	4	2
Back pain due to injury	1		Allergy	2	7
Burn		4	Back pain non-injury	6	2
Choking		1	Bleed/epistaxis/blood loss	11	7
Extremity injury	7	4	Collapse/dizziness/vasovagal syncope	91	82
Face injury	6		Epilepsy/seizures	18	20
Fracture	3	3	Fever/febrile convulsion		6
High energy trauma	1	2	Hart disease/ACS^c^/chest pain/palpitations/AP^d^	43	66
Hypothermia	1		Headache	2	1
Inhalation trauma	1	2	Hyperventilation	15	22
Injury due to accident/low energy trauma	63	75	Hypo- of hyperglycaemia	7	9
Injury due to chemical substance		1	Malaise	3	4
Injury due to electricity		1	Non specified neurologic complaints	1	2
Injury due to fall	94	57	Pain	5	4
Injury due to traffic accident	2	5	Pregnancy/parturition		2
Injury due to violence	2	5	Psychiatric/social complaint		4
Intoxication	3	17	Respiratory distress/shortness of breath/COPD^e^	19	20
Luxation/distortion	7	4	Resuscitation	9	7
Neck injury	4	5	Sepsis	1	
Neurological trauma	1	1	Shock		1
Suicide attempt	11	11	Stroke/TIA^f^	11	17
Suturing	2		Subdural bleed/subarachnoid haemorrhage	1	2
Wound/wound leakage	7	2	Thrombosis	1	
Deceased n (%)	3 (0,6 %)	1 (0,2 %)	Vomiting/abdominal pain/acute abdomen/vomiting blood	10	7

^a^ *n* = 12 missing excluded; ^b^ *n* = 3 missing excluded; ^c^ *ACS* acute coronary syndrome, ^d^ *AP* angina pectoris, ^e^ *COPD* chronic obstructive pulmonary disease, ^f^ *TIA* transient ischemic attack

### Diagnostic measurements

The monitoring of vital signs in patients is presented in Table 3. In general, diagnostic measurements according to the national EMS standard were applied by RNs and by PAs. PAs used the SCEBS methodology (16 %, *n* = 77) and reported on exams of organ tract systems in one third of the EPRs (31 %, *n* = 155). These reports were not identified for the RNs.

**Table 3:** Characteristics of diagnostic measurements of patients by PA (*n* = 2) and RN (*n* = 23)

	PA (*n* = 493)	RN (*n* = 498)
Monitoring vital sign of patient *n* (%)		
Respiratory rate	294 (60 %)	343 (69 %)
Oxygen saturation	219 (44 %)	246 (49 %)
Systolic blood pressure	229 (47 %)	269 (54 %)
Diastolic blood pressure	229 (47 %)	255 (51 %)
Pulse rate	316 (64 %)	325 (65 %)
ECG^a^/heart rhythm	39 (8 %)	144 (29 %)
GCS/AVPU^b^	430 (87 %)	437 (88 %)
Glucose	55 (11 %)	95 (19 %)
Body temperature	33 (7 %)	12 (2 %)
Pain intensity score	0 (0 %)	72 (15 %)
SCEBS^c^methodology used *n* (%)	77 (16 %)	0 (0 %)
Systematic physical exams of organ tract systems *n* (%)	155 (31 %)	0 (0 %)

^a^ *ECG* electrocardiograph, ^b^ *GCS/AVPU* Glasgow Coma Scale/Alert Voice Pain Unresponsive, ^c^ *SCEBS* Somatic complaints, Cognitions, Emotions, Behaviour, and Social functioning of the patient

### Interventions

In general, we found limited information on applied interventions in conjunction with the national EMS protocol, except for pharmacological interventions (Table 4). PAs provided medical advice to 48 % of their patients (*n* = 235). For RNs we found no reports on medical advice.

**Table 4:** Characteristics of interventions provided by PA (*n* = 2) and RN (*n* = 23)

	PA (*n* = 493)	RN (*n* = 498)
*Interventions described in the national EMS standard*		
Administer medication according to national EMS standard *n* (%)	84 (17 %)	87 (17 %)
Placement of intravenous drip *n* (%)	23 (5 %)	36 (7 %)
Supply of oxygen *n* (%)	14 (3 %)	5 (1 %)
Immobilisation *n* (%)	10 (2 %)	0 (0 %)
*Interventions not described in the national EMS standard*		
Suture *n* (%)	16 (3 %)	0 (0 %)
Administer medication not in national EMS standard *n* (%)	47 (10 %)	0 (0 %)
Medical advice to patient *n* (%)	235 (48 %)	0 (0 %)

### Outcome of emergency care

Table 5 shows the outcomes of emergency care. PAs completed their treatment on scene significantly more often than RNs. PAs referred 50 % (*n* = 245) of the patients to another health care professional, while RNs referred 73 % (*n* = 351) (*χ*^*2*^ = 52.9, df = 1, *P* <0.0001). In conjunction, we found that PAs consulted other health care professionals (GP, emergency physician, etc.) significantly more often compared to RNs (*χ*^*2*^ = 35.5, df = 1, *P* <0.0001), both consulted the GP most often. However, PAs and RNs referred more patients to the ED, and less patients to the GP. There was no significant difference between PAs and the RNs in length of treatment time on scene. After completion of the prehospital EMS care on scene, only a small proportion of patients contacted the dispatch centre again within 24 h (PA: 3 %, RN: 2 %) or within 72 h (PA: 5 %, RN: 4 %). This proportion was even smaller for the patients who were not referred to GP or ED and were only treated on scene by the PA or RN. Follow up contact after completion of prehospital EMS care showed no significant differences between PAs and RNs.

**Table 5:** Characteristics of care completion by PA (*n* = 2) and RN (*n* = 23)

	PA (*n* = 493)	RN (*n* = 498)	Differences between groups^a^
Referral after EMS treatment on scene	*n* = 489	*n* = 482	
*n* (%)	245 (50 %)	351 (73 %)	*P* <0.0001
In case of referral: Type of health care organisation referred to *n* (%)	*n* = 245	*n* = 351	
Referral to GP	44 (18 %)	86 (25 %)	NS
Referral to ED	201 (82 %)	265 (75 %)	
Consultation of health care professional	*n* = 493	*n* = 498	
*n* (%)	164 (33 %)	84 (17 %)	*P* <0.0001
In case of consultation: Type of health care professional consulted *n* (%)	*n* = 164	*n* = 84	
General Practitioner	119 (73 %)	73 (87 %)	
Emergency Department	18 (11 %)	2 (2 %)	*P* <0.05
Other (e.g. medical specialist)	27 (16 %)	9 (11 %)	
Length of treatment time on scene	*n* = 489	*n* = 488	
median in minutes (IQR)^b^	25 (19)	26 (17)	NS
Follow up contact after prehospital EMS care *n* (%)	*n* = 493	*n* = 493	
Within 72 h	25 (5 %)	20 (4 %)	NS
Within 24 h	16 (3 %)	12 (2 %)	NS
Follow up contact of non-referred patients (to ED/GP) after prehospital EMS care *n* (%)	*n* = 244	*n* = 129	
Within 72 h	9 (4 %)	4 (3 %)	NS
Within 24 h	7 (3 %)	2 (2 %)	NS

^a^ *NS* not statistically significant

## Discussion

The results of our study show that patients of PAs and RNs were comparable with respect to age, gender, and initial complaints/conditions. PAs and RNs reported diagnostic measurements according to the national EMS standard. In line with the medical education, PAs additionally used the SCEBS methodology (16 %), and a systematic physical exam of organ tract systems in a third of the patients. PAs and RNs provided similar interventions, as described in the national EMS standard. Additionally, the PA provided half of his patients with medical advice. Moreover, PAs showed significant differences in care outcome compared to the RN. PAs referred half of their patients to another physician, while RNs referred almost three out of four patients to a physician. The median treatment time of the PA and RN showed no variations. Finally, a small proportion of patients (4–5 %) called the dispatch centre within 72 h after completion of the emergency care on scene. Again, there were no significant differences between the PA and the RN.

Although it seems that PAs and RNs do not differ regarding their interventions according to the national EMS standard, it looks as if PAs thinks differently. PAs use a systematic physical exam more often, and consult other medical specialists more frequently. While RNs follow the national EMS standards and measure vital signs more often to get a complete picture of the patient. PAs are educated to use the SCEBS methodology, as a basis to decide on a preliminary diagnosis. Possibly the use of the SCEBS methodology makes additional measurement of vital signs superfluous. This might explain why PAs complete their treatment on scene more often, as they have more skills to perform a medical assessment on scene [3, 10], compared to RNs. Studies in other fields of healthcare have suggested that the basic competences of the PA for a defined group of patients are comparable to the competences of a physician such as the GP [11], ED physician [12], and surgical and anaesthesiology residents [13]. However, the PA needs a specific medical training, supervised by the GP, emergency physician or anaesthesiologist before these competences are gained. In our study the two PAs received previously an education as ambulance RN.

A previous Dutch study comparing solo emergency care provision of RNs with regular EMS treatment with a fully equipped ambulance team, showed that the solo emergency care provider was more likely to finish the treatment on scene [14]. In our study the PA treats even more patients on scene, and seems to operate from a more general medical clinical perspective, comparable to the approach of the GP.

The question remains, whether the quality of care provided by PAs is (at least) equal to the care provided by RNs. Based on this study, we only have limited information on the actual outcome of care. As we found no differences between PAs and RNs in treatment time and repeating contacts of the patients, one could suggest that more consultation with other medical specialists and less referrals of the PA did not result in an increase of additional contacts with ambulance EMS within 72 h. However, data on extra ED visits of the same group of patients were lacking. Therefore, it remains partially unknown to what extent the patients of PAs more often needed emergency care at a later stage, or even worse, developed adverse events. Furthermore, insight in cost analysis of the care of PAs versus RNs needs to be explored.

### Strengths and weaknesses

There are some limitations to this study. As we performed a retrospective document study, we did not observe the actual care provision of PAs and RNs. We based our findings on the EPR, and these data were not primarily gathered for research purposes. Therefore, the reliability of the results could be discussed. It is possible that not all emergency diagnostics and interventions, such as medical advice provided by the RN, e.g. ‘If the medication doesn’t result in adequate pain relief, please contact your general practitioner’, were documented in the EPR. Not all variables in our study concerned mandatory fields in the EPR. However, as the aim of our study was to provide insight in current patient care of PAs and RNs, we may argue that the report in the run sheets was not flattered in favour of research purposes.

The study period for the inclusion of patients of the PAs differed, as the PA of EMS VGGM finished his education 2 years later than the PA of EMS VRGZ. We chose to include the patients of the PAs at comparable levels of their experience, in order to provide a valid insight in the actual care provided to the patients.

The representativeness of results could be discussed, because we included patients of a limited number of two PAs. However, the actual employment of PAs in EMS in the Netherlands is relatively low (*n* = 12), so we included a 17 % sample for PAs. The yearly employment of RNs in 2014 was 2.180 [15], which means a RN study sample of approximately 1 %. Therefore, we assume that the study provides a limited, though adequate insight in the patient care of the PA as solo emergency care provider in EMS.

Patient care and outcomes of PAs and RNs are likely to be influenced by patient characteristics, initial conditions of the patient, and the preliminary diagnosis in the prehospital phase of emergency treatment. It seems unlikely that the dispatch centre caused selection bias, as the assignment of patients to the PA or RN is based on the distance between the patient on scene and the available solo emergency care provider. The dispatch centre does not take the type of patient or complaint into account. Unfortunately, the information on initial conditions provided by the dispatch centre is not organized according to a validated classification system. Therefore, it is unknown whether similarities and differences in initial complaints/conditions are in fact definition problems, or concern actual similarities and differences in patient groups between PAs and RNs. Furthermore, EMS lacks a validated classification system on preliminary diagnosis. Therefore, we were not able to provide insight into preliminary medical diagnosis related to the outcome of emergency care provision, such as referral to GP and ED. Future studies should address this issue, and examine whether the results, that the PA finishes more treatment on scene, could be influenced by potential differences in initial conditions and preliminary diagnosis of the patients.

### Future research

Despite the observation of these restrictions, the results of this first study on the role and function of the PA in prehospital EMS are very interesting and could be promising [16] regarding optimal care provision in prehospital emergency care. As PAs provide less health care referrals, this could lead to the prevention of (unnecessary) admissions to the hospital, potentially to a decrease of diagnostic measurements and interventions in the ED, and furthermore, could result in a cost reduction [17]. Areas for future research should be focused on external generalisation of study results, by scaling of the study design to a larger (national) level. Potential bias by differences in initial conditions and preliminary diagnosis of patients should be researched. Furthermore, the quality and outcome of emergency care provision on scene versus referral should be examined. Finally, cost analysis and cost effectiveness of the employment of the PA in ambulance EMS need to be further studied.

## Conclusions

This study described the patient care of PAs and RNs as solo emergency care provider in EMS. In line with the nursing education RNs and PAs performed diagnostic measurements and interventions according to the national EMS standard. In line with the medical education, the PA additionally used the SCEBS methodology and a systematic physical exam of organ tract systems. In the outcome of care, the PA completed the treatment on scene significantly more often, while the median treatment time of the PA was comparable to that of the RN. Furthermore, the PA consulted significantly more often other medical specialists, and provided half of his patients with medical advice. Patients of PAs and RNs did not differ regarding additional follow up contacts with the dispatch centre within 72 h after care completion on scene. The role and function of the PA in prehospital EMS could be promising regarding optimal care provision in prehospital emergency care.
